# A Mediterranean-Diet-Based Nutritional Intervention for Children with Prediabetes in a Rural Town: A Pilot Randomized Controlled Trial

**DOI:** 10.3390/nu14173614

**Published:** 2022-09-01

**Authors:** Isabel María Blancas-Sánchez, María Del Rosal Jurado, Pilar Aparicio-Martínez, Gracia Quintana Navarro, Manuel Vaquero-Abellan, Rafael A. Castro Jiménez, Francisco Javier Fonseca Pozo

**Affiliations:** 1 Emergency Department, Reina Sofia’s University Hospital, Andalusian Health Care System, 14004 Cordoba, Spain; 2 Grupo Investigación GC09 Nutrigenomics, Metabolic Syndrome, Instituto Maimónides de Investigación Biomédica de Córdoba (IMIBIC), Reina Sofia’s University Hospital, 14004 Cordoba, Spain; 3 Departamento de Enfermería, Farmacología y Fisioterapia, Campus de Menéndez Pidal, Universidad de Córdoba, 14071 Córdoba, Spain; 4 Grupo Investigación GC12 Clinical and Epidemiological Research in Primary Care, Instituto Maimónides de Investigación Biomédica de Córdoba (IMIBIC), Reina Sofia’s University Hospital, 14071 Cordoba, Spain; 5 Distrito Sanitario Córdoba Guadalquivir, Andalusian Health Care System, 14004 Cordoba, Spain

**Keywords:** child-nutrition disorders, prediabetic state, nutrition therapy, diet, Mediterranean, rural health

## Abstract

Prediabetes is a pathological condition in which the blood glucose concentration is higher than normal concentrations but lower than those considered necessary for a type 2 diabetes mellitus diagnosis. Various authors have indicated that the Mediterranean Diet is one of the dietary patterns with the most healthy outcomes, reducing high levels of HbA1c, triglycerides, BMI, and other anthropometric parameters. The main objective of this study was to determine the efficacy of the nutritional intervention for children with prediabetes, including the effectiveness of this nutritional education regarding anthropometric parameters. A randomized pilot trial with two groups, an experimental group (EG) and a control group (CG), using intervention in dietary habits with nutritional reinforcement was carried out on 29 children with prediabetes from a rural area. The nutritional intervention was analyzed through astrophotometric and glycemic measurements and validated surveys. Results: The results indicated improvement in eating habits, adherence to the Mediterranean diet, anthropometric measurements, mainly body mass index and perimeters, and analytical parameters, with a significant decrease in glycated hemoglobin in the EG compared to the CG (*p* < 0.001). Although the results showed that both groups’ anthropometric parameters improved, a more significant decrease was observed in the experimental group compared to the control.

## 1. Introduction

Nutrition can be defined as incorporating nutrients for adequate development and growth, nourishment, and metabolic balance [1]. During the last decades, there has been a switch to a higher intake of carbohydrates (45%), the majority being sucrose (27%) and lactose (27%), which has caused increases in body fat [2,3]. A healthy diet and suitable nutrition have been linked to gestational, perinatal, pediatric, and adult health, decreasing the probability of multiple acute and chronic diseases [4,5].

High body mass index (BMI), associated with overweight and metabolic disorders, is a risk factor for severe health problems during adulthood [6,7], such as cardiovascular diseases (CVDs) [8]. In 2009, the World Health Organization (WHO) indicated that one in ten children of school age (5–17 years) was overweight or obese [3,9]; the current rate is one out of five children [8,10,11]. It is currently estimated that in European countries, up to 20% of school-age children are overweight, and 5% are obese [12]. These rates and their rapid increase have been linked to an unbalanced diet and a sedentary lifestyle [13,14,15].

Children with high BMI have a greater risk of suffering from metabolic syndrome, a clinical entity that can damage the vascular endothelium, favoring the appearance of atherosclerosis and the subsequent development of CVDs [12,16,17,18]. Furthermore, this syndrome is a risk for future problems related to high glycated hemoglobin (HbA1c) values, increased insulin resistance, and changes in visceral fat [19,20]. High and borderline HbA1c levels, which can be used to define prediabetes, are under study in pediatric populations due to their predictive power [21,22].

Specifically, a concerning disorder increasing in adolescents and young adults is prediabetes [23]. Prediabetes is a metabolic disorder characterized by blood-glucose concentrations higher than those considered normal but not high enough to diagnose diabetes mellitus 2 (MD2) [24]. An American cross-sectional analysis from 2005 to 2016 indicated that the prevalence of prediabetes among adolescents was 18.0% [23], linked to higher BMI, low-density lipoprotein cholesterol levels, systolic blood pressure, and central adiposity and lower insulin sensitivity. By contrast, the prevalence of children with prediabetes was lower (around 2.51% globally, with a 95% confidence interval (IC) of 1.61–3.41%), and lower still in European countries (4.52%, IC 0.53–9.56%) and rural areas (2.47, 95% IC 0.93–4.02). However, this prevalence increased globally by 1.73% from 2011 to 2018 compared to the first decade of this century [25].

In children, prediabetes has been linked to age, gender, high BMI, family history of diabetes, low physical activity, living in an urban area, dietary habits, lifestyle, and sociodemographic and economic factors [25,26,27,28,29]. A recent study carried out in New Zealand indicated that the prediabetes rate (16%, with 6% in European children) was associated with ethnicity, anthropometric measures, and physical activity levels [30]. These results support the notion that anthropometric measures are critical in prediabetes and lifestyle [25,26,27,28,29,30]. Nutrition is understudied as a factor compared with other indices, such as BMI [27].

Various authors have indicated that the Mediterranean diet is one of the dietary patterns with the healthiest outcomes, reducing high levels of HbA1c, triglycerides, high BMI, and other anthropometric parameters [6,31,32,33,34]. The study Prevention with the Mediterranean Diet (PREDIMED) [6,31] concluded that up to 40% of the children studied reduced the appearance of MD2, without associated weight loss, after an intervention based on the Mediterranean diet. Other authors observed that a nutritional intervention based on adherence to the Mediterranean diet resulted in a significant reduction in weight in the experimental group (EG) compared to the control group (CG) [35]. Another study focused on determining the effectiveness of the vegetarian versus the Mediterranean diet highlighted that both diets effectively reduced body weight, body mass index, fat mass, and lipid profile [34]. Moreover, the Mediterranean diet has reduced the risk for various metabolic and endocrine diseases, such as diabetes or metabolic syndrome, preventing or delaying the progression to MD2 [35,36]. Additionally, compared to the healthy diet, based on the American Diabetes Association’s recommendation, the Mediterranean diet reduced HbA1c and triglyceridemia [37].

In Spain, a nutritional intervention based on the Mediterranean diet was shown to decrease BMI and reduce or maintain blood glucose control (reducing HbA1c 0.3–2.0% at three to six months in young adults) [36], thereby improving metabolic levels [32,38]. Nonetheless, the adherence to diets among people that suffer from high HbA1c levels is low [36]; consequently, it is relevant to improve its adherence, mainly via nutritional education, which is the most robust scientifically supported intervention [36]. However, few studies have addressed the efficacy of these interventions in prediabetes in areas with reduced access to health services or smaller populations, such as rural areas [39], and in children [25,32]. The only reported clinical trial focused on a healthy-lifestyle intervention in Spanish children, combining lifestyle education and a psycho-educational program, is the PREvention of DIabetes in KIDs (PREDIKID) study [40]. Nonetheless, this trial is based on the efficacy of exercise combined with lifestyle education and does not determine the effectiveness of the Mediterranean diet in reducing the HbA1c [40].

Therefore, the objectives of this study were as follows: to determine the efficacy of the nutritional intervention on children with prediabetes; to evaluate the improvement of an individualized and directed nutritional intervention compared to a standardized diet (an adapted Mediterranean diet vs. a healthy standardized diet according to the American Diabetes Association’s recommendation); and to determine the effectiveness of nutritional education regarding anthropometric parameters and adherence to a Mediterranean diet in children with prediabetes.

## 2. Materials and Methods

### 2.1. Study Design

This research focused on a pre-and post-nutritional, randomized, single-blind intervention on parallel groups of schoolchildren, EG and CG, with prediabetes in a rural area, with an average income per household of around EUR 19 thousand. 

The study design was divided into two phases, pre-and post-intervention, comparing a nutritional intervention based on the adapted catalog with recommendations of healthcare professionals with an adapted Mediterranean diet (EG) and a standardized healthy diet based on the current nutritional advice from healthcare centers (CG). In each phase, a follow-up was carried out with five visits (32 days apart), from the initial visit (v0) in weeks one and two, in which the study variables were gathered (anthropometric values and blood sample), to a final visit (v4), when the study variables were collected after the nutritional intervention. The study was approved by the Regional Ethical Committee, registered on the regional database (ID:2353) and at Clinical Trials (NCT05424107) (https://clinicaltrials.gov/ct2/show/NCT05424107?type=Intr&cond=PreDiabetes&cntry=ES&draw=2&rank=5, accessed on 21 June 2022).

### 2.2. Study Population, Sampling, and Data Collection

The study’s target population was a sample of primary- and secondary-school children presenting altered HbA1c levels [24]. The inclusion criteria were the presentation of HbA1c levels between 5.7 and 6.4% and informed and signed consent from the minor and the minor’s parents or guardians. The exclusion criteria were the presence of HbA1c levels below 5.7% or above 6.4%, pathological metabolic disease, such as diabetes or metabolic syndrome, and a lack of signed informed consent from the minor and their legal guardian.

The sampling was based on estimating high BMI in rural areas of Spain (12.6%), a confidence interval of 95%, and a precision of 4.5. The minimum sample was set as 233 children based on BMI, estimating 2.47 with a 95% IC 0.93–4.02 of the sample with prediabetes in rural areas [25].

The participants were recruited through non-probabilistic convenience sampling between March 2018 and February 2019. For the recruitment of the participants, informative sessions were held in the educational centers for legal guardians, parents, and participants. In these sessions, the study objective and planning were explained, along with the voluntary nature of their participation, and the questions or doubts raised by the tutors, parents, and pediatric patients were answered. After these information sessions, the last session was held before data collection. The interested parties signed the informed consent, which was ratified by the tutors, patients, and researchers who were present.

**Figure 1:**
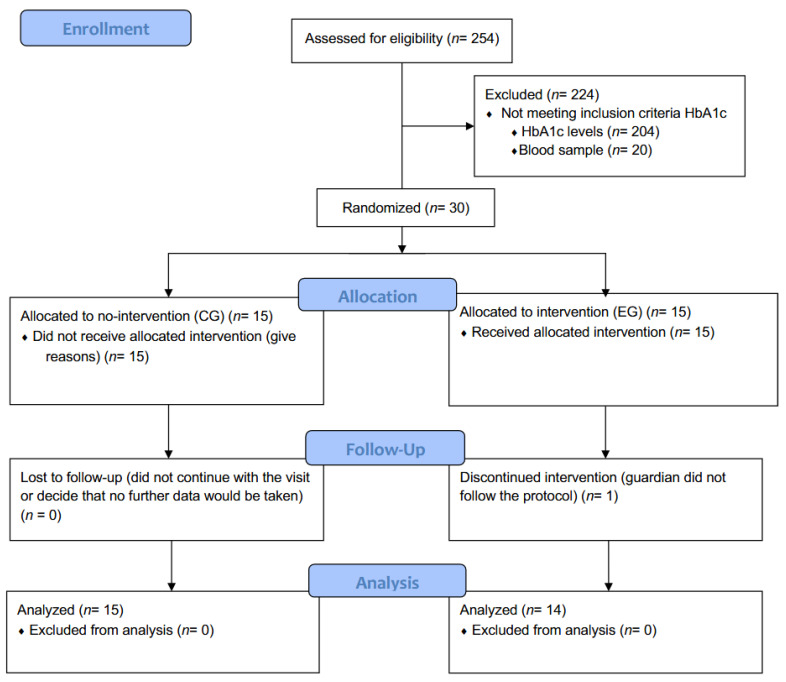
Flow diagram of the selection and randomization of the sample following the CONSORT guidelines.

Most of the participants (238 out of 254 (93.7%)) agreed to undergo blood tests measuring different analytical data, including HbA1c. Out of this sample, 30 students (8.46% of the total sample) met the inclusion criteria for this intervention, which matched the prevalence of children with prediabetes in European countries (4.52%, IC 0.53–9.56%) [25]. The 29 children included in the study were assigned in a simple random way to each of the two groups: 14 EG and 15 CG. This randomization was done by an outsider researcher via an online program based on their identification number (https://www.random.org/lists/ accessed on 1 January 2019). Such program allowed to the randomly assigned each child to permuted group. One child was not included because the guardians’ decision followed the CONSORT flow diagram [41] (Figure 1).

### 2.3. Nutritional Intervention and Evaluation

**Figure 2:**
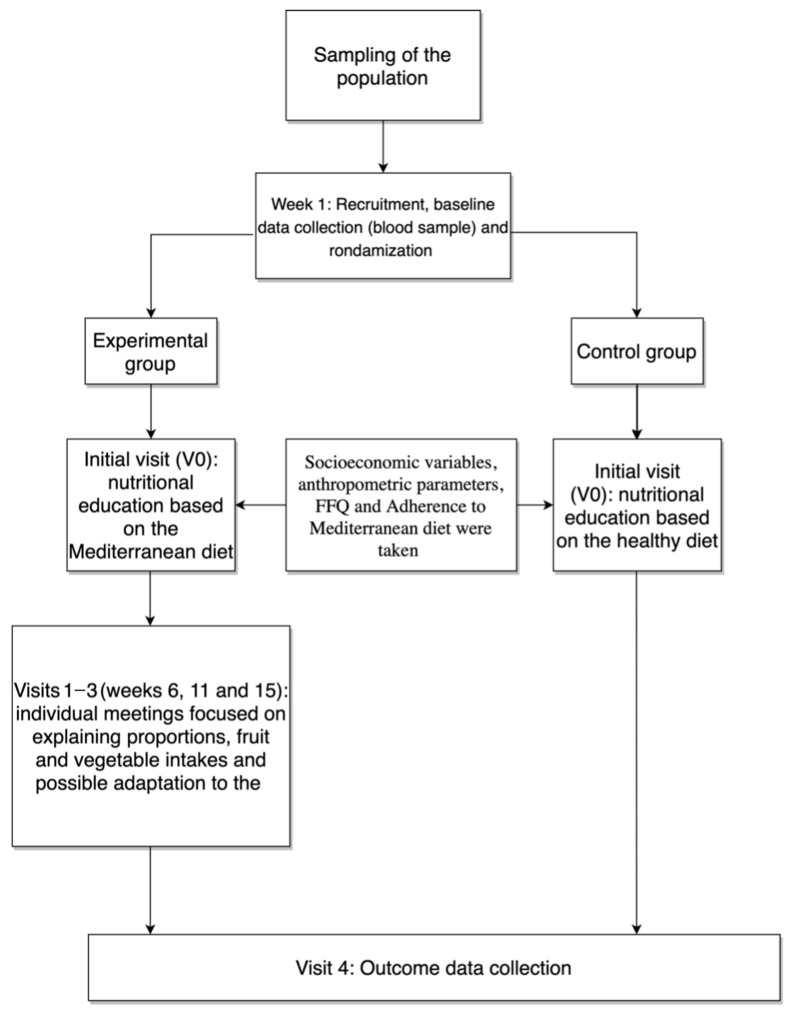
Flow chart of the nutritional intervention, visit follow-ups, and outcome data collection.

The nutritional intervention and the outcome data (anthropometric parameters, glycemic values, and questionnaire on adherence to the Mediterranean diet and food-consumption frequency) are described according to four visits distributed over six weeks. The structure of the nutritional intervention and planification implemented was as follows (Figure 2).

Visit 0 (weeks one and two): The initial one-hour visit was conducted individually on all study subjects (29 children). The anthropometric parameters were taken on these visits. The first assessment of the children’s eating habits and adherence to a Mediterranean diet was carried out. In addition, a socioeconomic evaluation was carried out on all the parents through an original and specific questionnaire consisting of nine questions. During the same visit, the EG received targeted nutritional education, with specific recommendations on the Mediterranean diet. On the other hand, the CG only received an information sheet with standardized recommendations for healthy eating, called the Decalogue of Healthy Eating.

Visits 1 (week six), 2 (week eleven), and 3 (week fifteen): These three visits took 30 min to complete. They included an evaluation of dietary adherence, the resolution of doubts about diet and food, and nutritional reinforcement for the children and families in the EG. Visit 4 (week twenty): The final evaluation was conducted for both groups’ children. The Mediterranean diet adherence questionnaires were filled out, and the anthropometric variables and blood samples were retaken.

### 2.4. Parameters and Measuring Instruments

The parameters measured were HbA1c levels, height (m^2^), weight (kg), waist circumference (cm), hip circumference (cm), arm circumference (cm), BMI (kg/m^2^), validated nutritional assessment questionnaire focused on adherence to the KIDMED, Food Frequency Questionnaire (FFQ), and the decalogue of healthy eating. All these measurements were taken up to six months from the V0. 

The anthropometric evaluation was carried out on all the study subjects, measuring the following parameters: height, weight, BMI, waist circumference, hip circumference, arm circumference, body fat percentage, and fat-free-mass percentage. The measurements obtained using a tape measure and Omron BF511 impedanciometry were grouped according to the recommendations of the World Health Organization.

Validated nutritional assessment questionnaires that focused on the frequency of food consumption, quality questionnaires, and adherence to the Mediterranean diet, such as the KIDMED, were applied. In addition, the decalogue of healthy eating was used to determine adherence to the change in diet.

The Food Frequency Questionnaire (FFQ) includes 137 foods classified into 14 groups (dairy products, eggs, meat or meat products, fish or shellfish, vegetables, potatoes, fruits, nuts, legumes, cereals, olive oil, pastries, cakes or sweets, and alcoholic beverages). Frequencies were recorded using a 9-category Likert-type scale (from “rarely” to “6 or more times per day”). In addition, the rate of consumption of common foods in this population group, such as juices (nectars, concentrates, etc.), sweets or sugary breakfast cereals (with chocolate, honey, etc.), was collected, along with the habitual culinary techniques used in the children’s houses. Energy and nutrient intakes were calculated using Spanish food composition tables.

The quality index of the KIDMED aimed at children and adolescents comprises 16 questions related to adherence to the Mediterranean diet and seven items complementary to the test. All questions were answered positively or negatively. Of the 16 main questions, question numbers 6, 11, 14, and 16, in their affirmative answers, had a negative meaning; therefore, they were worth (−1). On the other hand, the remaining questions whose affirmative answers represented a positive value concerning the Mediterranean diet were scored with (+1). Negative responses received no score (0). According to the test, the results were grouped into different levels of adherence to the Mediterranean diet: low (score 0 to 3), medium (score 4–7), and high (8 to 12).

Lastly, the decalogue of healthy eating, developed for the internal control of adherence to the prescribed dietary recommendations, allowed the dietitians and the children/parents to determine which nutritional aspects were quickest and simplest to improve. On the test, the results were grouped into different levels of adherence to the diet: low (score 0 to 3), medium (score 4–7) and high (8 to 10).

Furthermore, the educational material designed and prepared specifically for the intervention group included printed documents and PowerPoint presentations for the group visits, focused on food cards and hidden nutrient techniques, healthy menus, diabetes, and the decalogue of the Mediterranean diet.

### 2.5. Statistical Analysis

The descriptive analysis of the categorical variables was expressed as a percentage, and the quantitative variables were expressed through measures of central tendency (mean ± standard deviation and 95% confidence interval). The normalization test (Shapiro–Wilk) indicated normalization inside the sample (*p* = 0.24), although there were differences for the total sample (*p* < 0.05). Differences in continuous variables between groups (EG and CG groups) were analyzed by Student’s test or Mann–Whitney Student when adequate. Paired measurements, and categorical variables were analyzed using the chi-squared test. All statistical analyses were performed using the statistical package SPSS (version 28.0) for Windows.

### 2.6. Ethical Aspects

This research followed the Helsinki Code and the Principles of Biomedicine and obtained the approval of the Ethical Committee in the Regional Committee of the provincial Hospital (Number 2353).

## 3. Results

Twenty-nine children with prediabetes participated in the study, with a mean age of ten years, fifteen of whom were male (52% of the sample). The boys showed higher values than the girls in terms of weight (53.31 ± 11.8 males vs. 31.77 ± 3.57 females), height (149.4 ± 9.51 males vs. 134.4 ± 5.87 females), waist circumference (82.03 ± 8.67 vs. 60.82 ± 5.61), arm circumference (24.47 ± 3.06 vs. 18.57 ± 1.74), and BMI (23.6 ± 4.49 vs. 17.54 ± 2.09) (*p* < 0.01). Despite the initial differences regarding gender, there were no significant differences between girls and boys regarding the Hb1Ac (*p* = 0.085). Regarding the family characteristics, most of the guardians or parents were married (89.7%), with a primary education level (48.3%), a housewife (37.9%), with an 8-day shift (47.4%), with a period of unemployment between 6 months and one year (41.7%), with two dependents (62.1%) and received more than EUR 900 per month (62.1%).

Both groups (CG and EG) had no significant differences for the variables studied during the initial visit (V0) (Table 1). In this sense, the results of the glycemic profiling showed how both groups had similar values at the first visit (5.86 ± 0.13% in EG vs. 5.91 ± 0.18% CG; *p* = 0.47 for HbA1c and 12.07 ± 12.2 mIU/L in EG vs. 11.7 ± 7.2 mIU/L in CG; *p* = 0.72 for insulin) (Table 1).

The results of the intervention (Table 2) showed that both groups experienced significant decreases in waist, arm, and hip circumferences (*p* < 0.05). The results indicated no significant differences between the GE and GC regarding the decrease in the perimeters. Nonetheless, the children from the EG showed a more statistically significant decrease in waist circumference (−3.19 cm; *p* = 0.001 EG vs. −2.32cm; *p* = 0.02 CG) and hip circumference (−2.51 cm; *p* = 0.012 EG vs. −2.36 cm; *p* = 0.018 CG). Furthermore, the fat-free mass percentage increased after the intervention, and this increase was greater in the CG compared to the EG (2.98%; *p* = 0.003 in CG vs. 2.47%; *p* = 0.016 in EG). The results also showed that there was no significant difference after the nutritional intervention regarding BMI and body fat percentage (*p* > 0.05) (Table 2).

**Figure 3:**
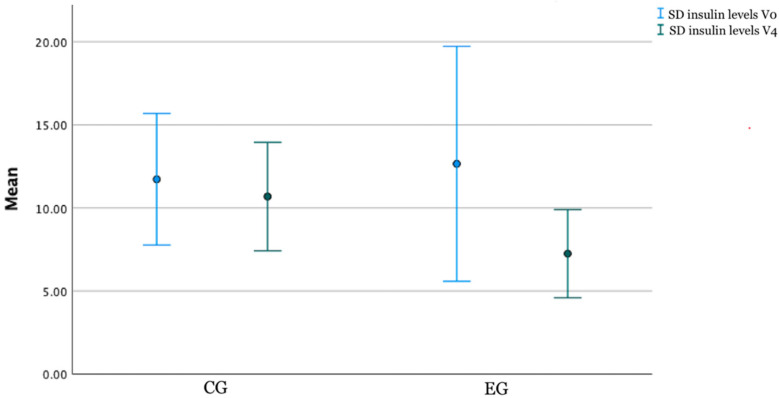
Changes in insulin levels after the interventions in each EG and CG (V4).

**Figure 4:**
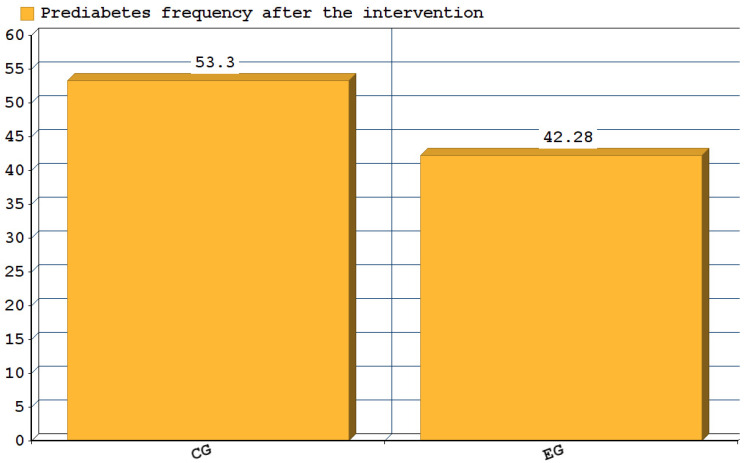
Prediabetes frequency after the intervention (V4) in the control and experimental group.

Another parameter that showed improvement after the intervention was HbA1c and insulin levels (Table 2). In both groups, HbA1c values decreased significantly between the first and last visit (*p* = 0.001 in CE and *p* < 0.001 in CG), but insulin had a significant change only in the intervention group (7.3 mIU/L; *p* = 0.006 in EG vs. 10.8 mIU/L; *p* = 0.41 in CG), indicating significant differences between EG and CG for insulin level (*p* = 0.046) (Figure 3). In addition, after the intervention, the frequency of HbA1c over normal values decreased significantly (58.85% in EG and 46.6% in CG) (Figure 4).

**Figure 5:**
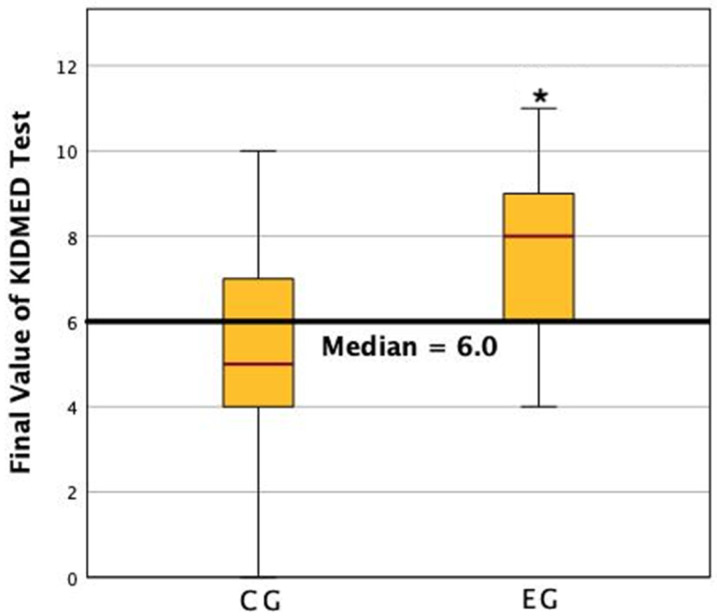
Changes in KIDMED between groups from the first visit to the last. An asterisk denotes a statistically significant difference between the interventions.

According to the KIDMED test, showed that 44.8% of the sample (*n* = 29) had low adherence to the Mediterranean Diet, 48.3% showed medium adherence, and only 6.9% showed good compliance with the healthy dietary model. The results of the KIDMED were 3.2 ± 2.1 (95% CI 2.01–4.39) for the CG and 4.57 ± 2.47 (CI 95% 3.14–5.99) for the EG, with no significant differences between groups at the beginning (*p* = 0.69). After the intervention, the results of the KIDMED test improved in both groups, significantly improving the EG and compared to CG (2.5; *p* = 0.012 EG vs. 1.89, *p* = 0.056 CG) (Figure 5). In this sense, after the intervention, the EG population went from having low adherence (42.9%) to acceptable adherence (only 14.3% showed low adherence).

Before the intervention, the mean energy intake of the 29 children with prediabetes was 2883 ± 430 kcal/day. The primary energy contribution in the diet was made by carbohydrates (50%, completes 27%, and simple 23%), followed by fats (35%, monounsaturated 17%, saturated 12%, and polyunsaturated 6%), and proteins (15%); the intake of carbohydrates and fats in the EG decreased after the intervention (*p* < 0.01). The FFQ indicated a significant decrease in food intake, mainly in terms of carbohydrates and fat, in both the EG and the CG (*p* < 0.01). Furthermore, the results after the intervention regarding the FFQ score showed that the children from the EG experienced a higher reduction in food intake (47.14; IC 95% 21.9–72.33 points in the FFQ; *p* < 0.001 for the children in the EG vs. 23.3; IC 95% 8.9–37.43 points in the FFQ; *p* = 0.002 children in CG). 

According to the adherence to the decalogue of healthy eating, variation in percentage concerning items included in the decalogue of the healthy eating questionnaire was shown (Table 3). The differences between visits in the consumption of vegetables and whole meal bread increased, and a decrease in the consumption of carbonated beverages was noted for both groups (*p* < 0.001) (Table 3). Although there were improvements in both groups (Table 3), the results for all the items indicated a significant improvement in the EG (0.8; *p* = 0.021) compared to the GC (0.4; *p* = 0.25). The EG children decreased their weekly consumption of red and processed meats by 70%; half of them did not consume carbonated and sugary drinks, industrial pastries, snacks, or sweets (Table 3). Finally, water became the main drink for 100% of the children (*p* < 0.05), indicating no significant differences between groups (*p* = 0.89) (Table 3).

## 4. Discussion

This pilot study analyzed the efficacy of a nutritional intervention based on the Mediterranean diet in comparison with a healthy standardized diet to reduce HbA1c and insulin levels and, therefore, reduce the prevalence of prediabetes in a rural pediatric population. Furthermore, this study analyzed the impact of nutritional interventions guided by health care providers to promote healthy habits and control risk factors, such as high BMI.

The initial results of the data indicated that the frequency of prediabetes in a rural town seemed to be in line with the data from European countries and lower-income areas [25]. These initial data indicate that the prevalence of prediabetes also increased and was more common in boys [25,26,27,28,29,30]. 

The results for both groups indicated that the BMI did not significantly decrease in the Mediterranean or healthy diet, which matched the results of several studies regarding the possibility of no significant decrease in weight or BMI [6,31,32,33,34]. However, the waist, arm, and hip diameters significantly improved in both groups; the decrease was more notable in the experimental group than in the control. These results coincide with those of Albert Pérez et al. (2018), which indicated that nutritional interventions based on diet or exercise decrease anthropometric values and metabolic parameters [42]. Additionally, both groups experienced an increase in fat-free percentage, with the increase higher in the CG, which was in line with a recent meta-analysis regarding prediabetes that indicated the positive impact of the Mediterranean diet and of healthy diets following the American Diabetes Association’s recommendations [37].

Moreover, the changes in the HbA1c in both groups highlighted how the Mediterranean diet and healthy diet, monitored by health care professionals, are effective interventions to control and reduce the risk of MD2. These results also matched the findings of the last executive summary of the Spanish Atherosclerosis Society [36], which highlighted that the Mediterranean diet and healthy diets based on the American Diabetes Association’s recommendations (mainly the Dietary Approaches to Stop Hypertension, also known as DASH) reduce HbA1c levels and risk factors in adults. The only parameter that differed between the groups was insulin levels, which were related to insulin resistance. This seems to match the results of a previous analysis that indicated how the Mediterranean diet results in a modification in compound-specific gene expression via the involvement of the nuclear high-mobility group A1 (HMGA1) protein [43,44]. These results imply that the Mediterranean diet seems to provoke modifications in metabolic complications in humans, children, or adults, reducing pro- and anti-inflammatory adipocytokines and insulin resistance [44].

Another exciting result was the improvement in adherence to the Mediterranean diet and the modifications in food consumption. Both groups experienced a reduction in food intake, with a corresponding reduction in energy, possibly associated with the lessening of the anthropometric parameters and improvements in Hb1Ac. However, the children from the EG experienced a significantly decreased frequency of low adherence to the diet and increased consumption of healthy foods, with a simultaneous increase in Mediterranean diet adherence. Moreover, after the intervention, the five most frequently consumed foods among our study population were carbonated and sugary drinks, skim milk products, whole-milk products, vegetables, and fruits. The pre-intervention data coincided with a previous analysis in Croatia, which reflected how children (8 and 9 years old) have low adherence to the Mediterranean diet, with their consumption of processed and sugary foods at levels higher than those recommended, which can lead to insulin resistance and other health issues [25,36,45]. However, the post-intervention food preferences in this study were opposed to those presented in the ANIBES Study [46], in which the five most frequently consumed foods were bread, pastries, meats, olive oil, and sausages, suggesting that other factors, such as the role of health care professionals, intervene in food-intake changes. In this sense, the study by Costarelli et al. reflected the efficiency of nutritional interventions in schools, although adherence to the Mediterranean diet is related to the profiles of those who implement these interventions [47]. When teachers oversee interventions based on improving adherence to the Mediterranean diet, the results of the control and experimental groups do not differ significantly. 

Despite the differences regarding the children’s preferences, these previous findings [25,36,45,48] highlight that the low adherence to this diet is one of the factors that are increasing the prevalence of diabetes and other issues [25,26,27,28,29,30], reflecting how a nutritional intervention, such as that presented in the current study, with follow-ups by health professionals, can improve adherence, decrease inadequate food intake, and improve glycemic values. 

### Limitations, Implications for the Field, and Future Research

As with any research, this study has limitations. The major limitation was the sample size of 29 individuals, 15 in the CG and 14 in the EG, resulting from the study’s voluntariness and the established inclusion criteria. This limitation implies that the findings may be exaggerated. It is, therefore, relevant to conduct this type of analysis with a larger sample. Another limitation was related to the coexistence of other health programs carried out in schools; furthermore, sociodemographic and individual conditions need to be considered since income or parental status may have modified the food preferences and the effectiveness of the results. In this sense, since the groups were located in a rural area of Southern Spain, the results could be limited in their generalizability to other children with prediabetes, even with similar-sized samples. 

Despite these limitations, the presented results showed how the eating habits of children with prediabetes improve with a dietary intervention based on nutritional education that allows them to discern unhealthy consumption. The eating habits improved exponentially in both groups, particularly through the adaptation to and inclusion of the Mediterranean diet, indicating that the role of healthcare specialists in any nutritional intervention results in improved healthy eating [32]. Given the results, it would be interesting to establish a targeted dietary intervention with consecutive personalized visits by specialized health personnel at the primary care level and even in schools.

Additionally, since there is currently a lack of studies aimed at the pediatric population [42], this study shows that nutritional intervention effectively reduces glycemic parameters and, therefore, the prevalence of prediabetes in the child population and values related to insulin resistance decrease accordingly. These findings suggest that if an adequate strategy is carried out in dietary re-education from an early age through educational programs, such as the Food, Nutrition, and Gastronomy Program for Early Childhood Education (PANGEI) [47], the establishment of healthy habits from early infancy improves quality of life and seems to ensure proper development [32,49]. Moreover, recent studies show how nutritional interventions are effective in this population, maintaining these dietary changes over time (minimum 36 months), but few include biochemical parameters aimed at prediabetes [42]. 

For this reason, future research will be focused on a nutritional intervention carried out by health care professionals (such as nurses) with school-based follow-ups, monitored by a nutritionist with a larger sample of children and during a minimum period of 36 months.

## 5. Conclusions

This pre-and post-intervention research aimed at a rural pediatric population with levels close to diabetes showed how the Mediterranean diet effectively reduces biochemical and anthropometric parameters. This efficacy was measured through a nutritional evaluation using validated questionnaires, visits to the children to improve their eating habits, and their adherence to the Mediterranean diet.

## Figures and Tables

**Table 1:** The study included anthropometric characteristics of the 29 children with prediabetes according to the randomization group (initial visit (V0)).

Variables	Control Group (CG) (*n* = 15)	Experimental Group (EG) (*n* = 14)	Differences between GC vs. GE (*p*-Value)
Gender (boys)	7 (46.7%)	8 (57.1%)	0.6
Weight (kg)	41.12 ± 12.04	44.18 ± 16	0.36
Height (m)	140.95 ± 9.29	143.2 ± 27.4	0.25
BMI ^1^ (kg/m^2^)	20.2 ± 4.1	20.9 ± 4.3	0.42
Waist circumference (cm)	68.9 ± 12.8	74.8 ± 15.3	0.4
Arm circumference (cm)	21.3 ± 3.2	21.9 ± 4.7	0.13
Hip circumference (cm)	88.9 ± 20.3	85.8 ± 12.5	0.51
BF% ^1^	29.9 ± 2.2	31.5 ± 3.9	0.58
FF% ^1^	24.3 ± 7.9	25.3 ± 7.9	0.41
Hb1Ac	5.86 ± 0.2	5.91 ± 0.1	0.47
Insulin level	11.72 ± 7.16	12 ± 10.01	0.68

^1^ BMI: Body mass index; BF%: Body fat percentage; FF%: Fat-free percentage

**Table 2:** According to the randomization group, values after the nutritional intervention of 29 children with prediabetes.

Variables	Control Group (CG) (*n* = 15)	Differences between V0 and V4 (*p*-Value) ^a^	Experimental Group (EG) (*n* = 14)	Differences between V0 and V4 (*p*-Value)	Differences GC vs. GE (*p*-Value) ^b^
Weight (kg)	40.27 ± 11.64	−0.85 (0.23)	42.97 ± 15.87	−1.83 (0.13)	0.46
Height (m)	142.45 ± 10.17	1.5 (0.85)	144.73 ± 13.39	1.5 (0.75)	0.91
BMI ^1^ (kg/m^2^)	19.58 ± 4.07	−0.62 (0.19)	20.61 ± 4.4	−0.69 (0.13)	0.62
Waist circumference (cm)	66.48 ± 12.74	−2.32 (0.02)	71.61 ± 13.65	−3.19 (0.001)	0.58
Arm circumference (cm)	18.94 ± 3.06	−2.36 (0.018)	19.39 ± 4.7	−2.51 (0.012)	0.45
Hip circumference (cm)	88.77 ± 23.32	−0.13 (*p* < 0.001)	85.42 ± 18.25	−0.38 (<0.001)	0.17
BF% ^1^	29.59 ± 3.11	−0.31 (0.39)	31 ± 3.71	−0.5 (0.22)	0.26
FF% ^1^	27.28 ± 8.9	2.98 (0.003)	27.77 ± 10.87	2.47 (0.016)	0.82
Hb1Ac	5.56 ± 0.2	−0.3 (<0.001)	5.51 ± 0.1	−0.27 (0.001)	0.51
Insulin (mIU/L)	10.8 ± 5.89	−0.8 (0.41)	7.3 ± 4.59	−4.8 (0.006)	0.046

^a^ The Student’s t for related samples and the Wilcoxon test were applied according to the normalization of the samples. ^b^ The Student’s t for independent samples and Mann–Whitney U test were applied to the normalization of the samples. ^1^ BMI: body mass index; BF%: body fat percentage; FF%: fat-free percentage

**Table 3:** Percentage of compliance with each item included in the decalogue of healthy eating after visit 4.

Items	CG		Differences between Visits	GE		Differences between Visits	Differences between GC and GE per Items
	Initial Visit	Last Visit		Initial Visit	Last Visit		
Consume ≥ 2 servings of vegetables per day, one of them raw	8.9%	67.7%	<0.001	7.1%	80%	<0.001	0.41
Use 3–4 tablespoons of extra virgin olive oil raw and for cooking	68%	80%	0.25	78.6%	100%	0.014	0.02
Eat ≥ 3 whole servings of fruit per day	4.3%	21.12%	0.02	7.1%	30%	0.016	0.158
Consume natural juices or smoothies	6.7%	33.3%	0.02	0%	80%	<0.001	<0.001
Preferably consume more whole grains (bread, pasta, and rice) than refined ones	0%	57.85	<0.001	0%	70%	<0.001	0.16
Eat ≥ 3 servings of legumes per week	45%	68%	0.04	50%	90%	0.004	0.014
Eat ≥ 3–4 handfuls per week of natural nuts	16.3%	36.7%	0.016	14.3%	50%	<0.001	0.045
Consume ≥ 3 servings of oily and/or white fish per week	48%	80%	<0.001	42.9%	80%	<0.001	0.45
Preferably consume lean meats without skin (chicken, turkey, rabbit, and pork tenderloin)	65%	73.3%	0.16	64.3%	100%	<0.001	0.012
Do not consume red and/or processed meats weekly (ribs, chops, hamburger, commercial sausages, etc.)	23.3%	66.7%	0.002	0%	70%	<0.001	0.16
Do not consume carbonated and/or sugary drinks weekly	13.3%	43.3%	<0.001	7.1%	50%	<0.001	0.84
Do not consume industrial pastries. treats and snacks weekly	0%	43.3%	<0.001	0%	50%	<0.001	0.049
Consume quality and minimally processed dairy products (natural yogurt, fresh cheese, etc.)	0%	13.3%	0.04	0%	20%	0.02	0.05
Water is the main daily drink	60%	100%	0.004	71.4%	100%	0.01	0.89

## Data Availability

The data is available via contacting the corresponding author.
